# The octanol/water distribution coefficients of ardipusilloside-I and its metabolites, and their permeation characteristics across Caco-2 cell monolayer

**DOI:** 10.1186/s13065-016-0175-y

**Published:** 2016-05-05

**Authors:** Wei-yu Cao, Bin Feng, Li-fei Cheng, Ying Wang, Ji Wang, Xiao-juan Wang

**Affiliations:** State Key Laboratory of Military Stomatology & National Clinical Research Center for Oral Diseases & Shaanxi Engineering Research Center for Dental Materials and Advanced Manufacture, Department of Pharmacy, School of Stomatology, The Fourth Military Medical University, Xi’an, 710032 Shaanxi China

**Keywords:** Ardipusilloside-I, Metabolites, LogP, Caco-2 cell monolayers, Intestinal absorption

## Abstract

**Background:**

Ardipusilloside-I (ADS-I) is a triterpenoid saponin extracted from Chinese medicinal herb *Ardisiapusill* A. DC. Previous studies have demonstrated the potent anti-tumor activities of ADS-I both in vitro and in vivo, and its main metabolites (M1 and M2) from human intestinal bacteria. However, the physicochemical properties and intestinal permeation rate of ADS-I and its metabolites are not understood. In this study, the octanol/water distribution coefficients (logP) of ADS-I and metabolites were investigated using standard shake flask technique, and their permeability properties was investigated across Caco-2 cells monolayer.

**Results:**

The logP of ADS-I, M1 and M2 was −0.01, 0.95 ± 0.04, 1.57 ± 0.11, respectively. The P_app_ values of ADS-I, M1 and M2 (in 10 μmol/L) across Caco-2 cell monolayers from the apical (AP) to basolateral (BL) direction were 1.88 ± 0.21 × 10^−6^ cm·s^−1^, 4.30 ± 0.43 × 10^−6^ cm·s^−1^, 4.74 ± 0.47 × 10^−6^ cm·s^−1^, respectively.

**Conclusion:**

Our data indicated that ADS-I has the poorer intestinal absorption than its metabolites (M1 and M2) in these experimental systems, suggesting that the metabolites of ADS-I may be the predominant products absorbed by the intestine when ADS-I is administered orally.

## Background

**Fig. 1:**
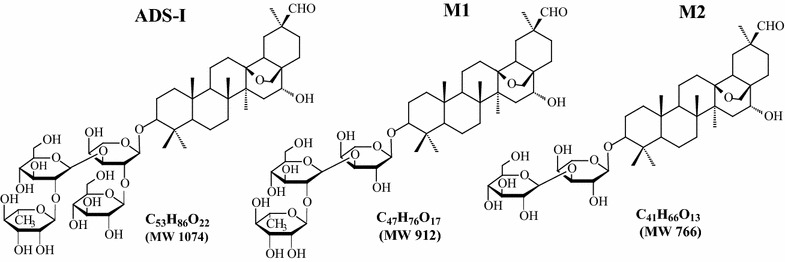
Chemical structures of ADS-I and its metabolites (M1, M2)

Ardipusilloside-I (ADS-I) [1] is a major bioactive triterpenoid saponin isolated from Chinese medicinal herb *Ardisiapusill* A. DC (*Mysinaceae*). The anti-tumor activity of this compound was first reported by Dr. Wang’s group [2], followed by many preclinical studies, showing that ADS-I induces tumor cell apoptosis and inhibits tumor cell growth, invasion and metastasis both in vitro and in vivo [3–6]. Pharmacokinetic study of ADS-I in rats shows that this compound has a poor intestinal absorption and the oral bioavailability [7]. Recently, we have shown that ADS-I could be mainly metabolized by human intestinal bacteria to metabolite M1 and M2 as shown in Fig. 1, in which the main metabolic pathway is deglycosylation of ADS-I through stepwise cleavage of sugar moieties [8]. These findings imply that these metabolites of ADS-I may be the primary active substances for its inhibitory activity against the growth of the tumor in vivo after oral administration, which however remains unknown. It has been known that the biological activities of drugs depend not only on their chemical structures, but also on their degree of lipophilic and membrane permeation that facilitate them across the cell membrane [9]. Although a previous study has revealed the poor intestinal absorption as well as a low oral bioavailability of ADS-I [7], the oral pharmacokinetic properties of ADS-I and its metabolites in humans have not investigated of yet. Evidence in literature indicates that deglycosylation of ginsenoside by intestinal bacteria to deglycosylated metabolites results in enhancing the permeability of the intestine, better adsorption into systemic circulation and exerting pharmacological effects of the ginsenoside [10–12]. Thus, we speculated that the deglycosylation of ADS-I to the metabolites (M1 and M2) might mediate the intestinal absorption of ADS-1 for increased oral bioavailability.

Lipophilicity, commonly expressed as octanol/water distribution coefficient (log P), has been considered as one important measurement of drug physicochemical parameters in drug discovery process to predict the pharmacokinetic properties and intestinal absorption of a drug [13, 14]. Caco-2 cell monolayer has been widely accepted as a standard in vitro model for prediction of drug absorption across human intestine and for mechanistic studies of intestinal drug transport since these cells show morphological and functional similarities to human small intestinal epithelial cells [15–17]. The objective of this study was designed to measure the logP of ADS-I and its metabolites using standard shake flask technique, and their permeability properties using Caco-2 cells monolayer. Thus, we could determine whether the products of ADS-I biotransformation by human intestinal bacteria played a role in the membrane permeability of ADS-I in human intestine.

## Results and discussion

### LogP of ADS-I and its metabolites in octanol/water

The LogP of ADS-I and its metabolites were determined by using standard shake flask method and HPLC-ELSD technique. The logarithm of logP values of ADS-I and its metabolites were shown in Table 1, indicating that the logP values of ADS-I, M1 and M2 were −0.01, 0.95 ± 0.04, 1.57 ± 0.11 respectively. According to a previous study [18], the value of logP from one to three suggests that the drug is easily absorbed by the intestine, and below zero poorly absorbed. Thus, our experiment results indicated that ADS-I was difficult or less to be absorbed, whereas M2 had the highest absorption. Furthermore, the data also showed that there was a correlation between molecular weight and lipophilicity of ADS-I and its metabolites. As a matter of fact, the absorption extent of these three compounds was negatively correlated with their molecular polarity and molecular weight. Indeed, the larger the molecular polarity and molecular weight of a compound, the more difficultly it is absorbed [19, 20]. ADS-I has the highest polarity and molecular weight compared to M1 and M2, the lower logP value of ADS-I suggests that this compound has a poor absorption in human intestine after oral administration. These results were in agreement with the low oral bioavailability of ADS-I in rats as reported previously [7], and may suggest that these metabolites may have better absorption than ADS-I.

**Table 1:** The octanol/water partition coefficients (logP) of ADS-I and its metabolites in phosphate-buffers (pH = 7.4)

ADS-I and its metabolites	MW (g/mol)	Log P (Mean ± SD)
ADS-I	1074	−0.01
M1	912	0.95 ± 0.04
M2	766	1.57 ± 0.11

Date are presented as mean ± SD (n = 3)

### The permeation characteristics of ADS-I and its metabolites across Caco-2 cell monolayer

#### Cytotoxicity assay

**Fig. 2:**
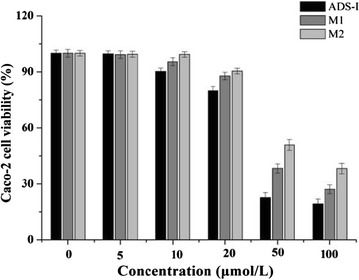
Cytotoxicity of ADS-I, M1 and M2 on Caco-2 cell monolayers using the MTT assay. Data are expressed as mean ± SD (n = 3)

The viability of cells was measured using MTT assay to evaluate the cytotoxicity of ADS-I and its metabolites (M1, M2) in Caco-2 cells prior to transport experiments. Generally, cell viability of more than 90 % indicated that the compounds at the stated concentrations were nontoxic to the cells [21]. As shown in Fig. 2, ADS-I, M1 and M2 at 0–10 µmol/L were nontoxic to the Caco-2 cells after incubation for 4 h. Therefore, 2, 5 and 10 µmol/L of each compound was used for two-way transport experiments.

#### Characters of Caco-2 cell monolayer

**Fig. 3:**
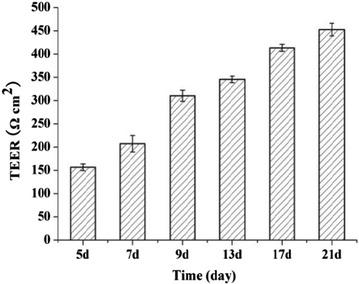
TEER values of Caco-2 cell monolayers at different time points. Data are represented as mean ± SD (n = 3)

**Fig. 4:**
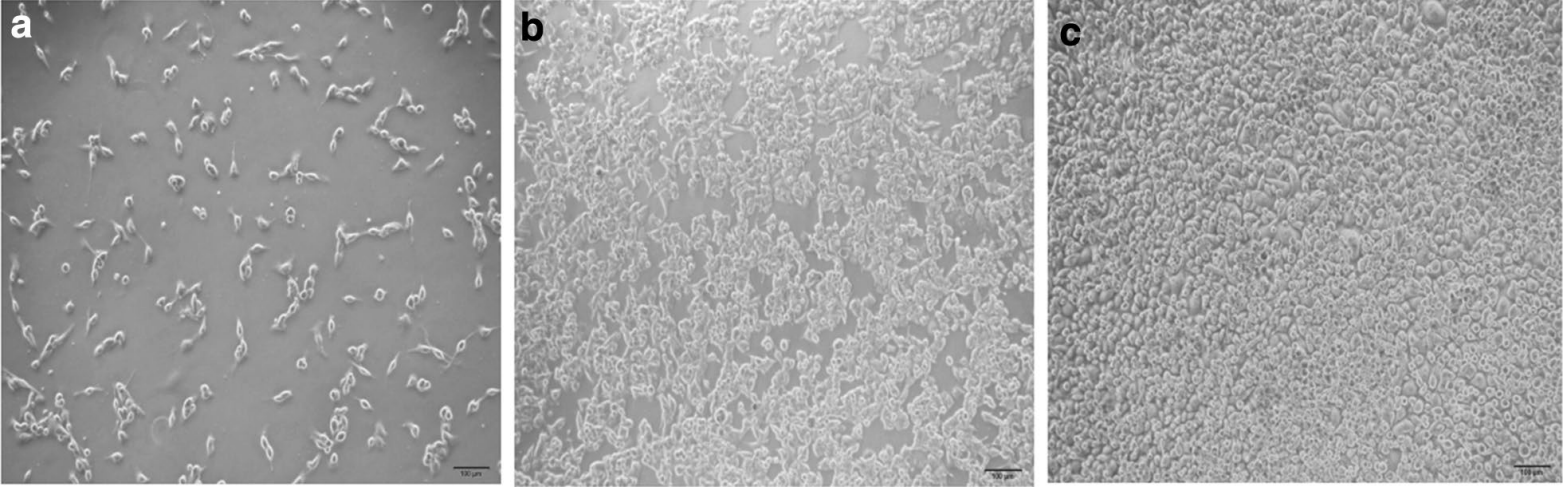
Caco-2 cell morphology, **a** the 2nd day; **b** the 7th day; **c** the 21st day

In order to confirm if the cells in culture formed a monolayer, the TEER values of the Caco-2 cell monolayer were measured at 5, 7, 9, 13, 17, 21 days after seeding, respectively. As shown in Fig. 3, the Caco-2 cell monolayer was completely formed on day 21 with TEER values above 400 Ω cm^2^ and was used for the transport experiments. In addition, electron microscope revealed the intact tight junctions in the Caco-2 cell monolayer (Fig. 4). Thus, the Caco-2 cell monolayer model established herein was validated for the permeation experiment.

#### Two-way transport of ADS-I and its metabolites across Caco-2 cell monolayer

**Fig. 5:**
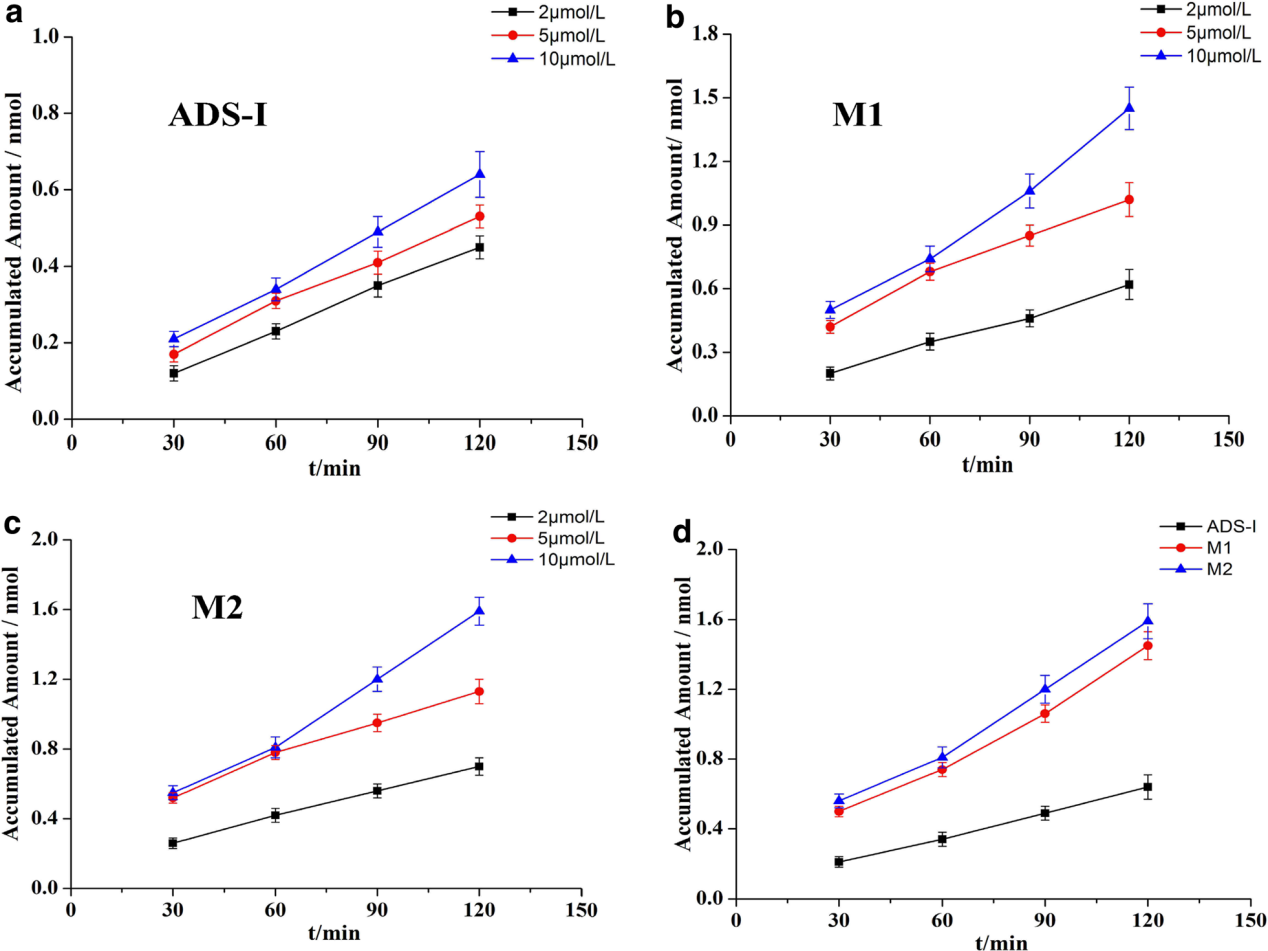
Change of accumulated amount of **a** different concentrations of ADS-I, **b** different concentration of M1, **c** different concentration of M2**, d** ADS-I, M1, M2 (10 µmol/L) across Caco-2 cell monolayer from AP to BL direction. Date represent the mean ± SD from three replicates

**Fig. 6:**
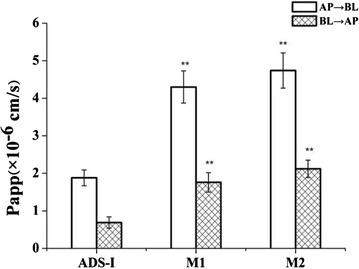
The P_app_ values of ADS-I, M1 and M2 (10 μmol/L) across Caco-2 cell monolayer from AP to BL direction and vice versa in 120 min. Date are presented as mean ± SD (n = 3). ^**^ *P* < 0.01 compared to the control group (ADS-I)

The permeability change of ADS-I, M1, or M2 across the Caco-2 cell monolayer at different concentrations from AP to BL direction was shown in Fig. 5, and the accumulated transfer amounts of ADS-I, M1 or M2 increased with a prolonged time of incubation as illustrated in Fig. 5a–c. With the same concentration, the flux amounts of M1 and M2 in AP to BL direction were similar, and both were greater than that of ADS-I (Fig. 5d). As shown in Fig. 6, the P_app_ value of ADS-I (10 µmol/L) was 1.88 ± 0.21 × 10^−6^ cm·s^−1^ for AP to BL direction, and was 0.69 ± 0.15 × 10^−6^ cm·s^−1^ for BL to AP direction in 120 min, which was considered to have a poor permeability and absorption rate similar to in vivo [7]. However, with stepwise removal of glycosyl groups in the metabolites (M1 and M2), the P_app_ values of these compounds increased and were higher than those of ADS-I across the Caco-2 cell monolayer in both directions. The P_app_ values of M1 (10 µmol/L) in the direction of AP - BL and BL - AP were 4.30 ± 0.43 × 10^−6^ cm·s^−1^ and 1.76 ± 0.26 × 10^−6^ cm·s^−1^ respectively, and of M2 (10 µmol/L) were 4.74 ± 0.47 × 10^−6^ cm·s^−1^ and 2.12 ± 0.23 × 10^−6^ cm·s^−1^ respectively. These data suggested that metabolites M1 and M2 were easier to be absorbed than ADS-I by the intestine.

The P_app_ values of ADS-I, M1 or M2 at different concentrations across the Caco-2 cell monolayer in both directions were shown in Table 2, indicating that the P_app_ values of these compounds gradually decreased with the increase of their concentration respectively. According to the formula (“The two-way transport experiment” section), the transport amounts of ΔQ increased along with Δt at the same concentration (C_0_), but the increased speed of ΔQ became slower along with the Δt, which meant that the value of ΔQ/Δt relatively decreased, thus the apparent permeability coefficient (P_app_) was also reduced. We inferred that the transport of ADS-I, M1 and M2 across the Caco-2 cell monolayer partially depend on their concentration, and some carrier might participate in the process of transportation. Besides,the P_app_ values of ADS-I and its metabolites (M1 and M2) in AP to BL direction were greater than that in BL to AP direction across the Caco-2 cell monolayer, and efflux ratio (ER) were all 0.3–0.6. These data suggested that ADS-I and its metabolites (M1 and M2) could be absorbed across intestinal epithelial cells in a passive absorption pattern, and the transport processes of these compounds might not be the substrate of apical efflux transporters.

**Table 2:** The P_app_ values of ADS-I, M1 and M2 at different concentrations across the Caco-2 cell monolayer in both directions in 120 min

Compound	Concentration (µmol/L)	Papp/× 10^−6^ cm·s^−1^		Efflux ratio
		AP → BL	BL → AP	
ADS-I	2	6.66 ± 1.32	2.50 ± 0.53	0.38
	5	3.10 ± 0.33	1.66 ± 0.24	0.54
	10	1.88 ± 0.21	0.69 ± 0.15	0.37
M1	2	9.13^**^ ± 1.65	3.14^**^ ± 0.58	0.34
	5	6.07^**^ ± 0.74	3.45^**^ ± 0.67	0.57
	10	4.30^**^ ± 0.43	1.76^**^ ± 0.26	0.41
M2	2	10.48^**^ ± 1.21	4.53^**^ ± 0.57	0.43
	5	6.76^**^ ± 0.85	2.60^**^ ± 0.43	0.38
	10	4.74^**^ ± 0.47	2.12^**^ ± 0.23	0.45

Date are presented as mean ± SD (n = 3). ^**^ *P* **<** 0.01 compared to control group (ADS-I)

It has been known that compounds with P_app_ values less than 2.0 × 10^−6^ cm·s^−1^ are considered to have a low absorption (0–20 %), while those with P_app_ values between 2.0 × 10^−6^ cm·s^−1^ and 10 × 10^−6^ cm·s^−1^ are considered to have a moderate absorption (20−70 %), and those with P_app_ values of higher than 10 × 10^−6^ cm·s^−1^ are considered to have a high absorption (70–100 %) [22]. In this study, the transport of the major metabolites (M1, M2) of ADS-I was compared with the parent compound ADS-I in the same system, and showed that ADS-I is a poorly absorbed compound, M1 and M2 belong to the moderately absorbed compound.

### Structure-intestinal permeability relationship

**Fig. 7:**
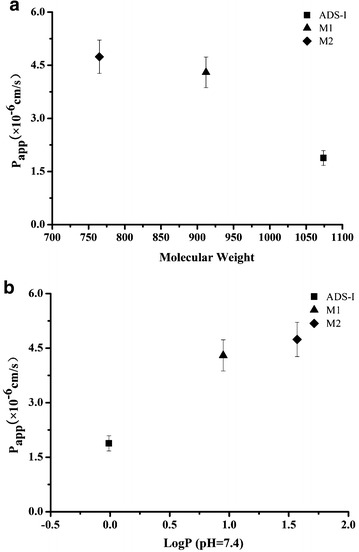
The apparent permeability (P_app_) vs. molecular weight (**a**), and logP (**b**) of ADS-I, M1 and M2 from the apical to basolateral direction across Caco-2 cell monolayer. Values are represents mean ± SD (n = 3)

Physicochemical characters, such as log P, log D and polar surface area, are generally measured for the prediction of drug permeability. To study the structure-intestinal permeability relationship, P_app_ values of ADS-I and its metabolites (M1 and M2) across Caco-2 cell monolayer were compared with their logP and molecular weight. In Fig. 7a, the P_app_ values of ADS-I and its metabolites (M1 and M2) transported from apical to basolateral direction (C_0_ 10 μM) were plotted as a function of their molecular weight, with stepwise removal of glycosyl groups in the metabolites (M1 and M2), the P_app_ values of these compounds increased and were higher than the parent compound ADS-I. Figure 7b showed the relationship between P_app_ and logP of ADS-I and its metabolites (M1 and M2), the P_app_ (AP to BL direction) values increased with an increase of LogP. These findings indicated that ADS-I and its metabolites (M1 and M2) across Caco-2 cell monolayer were well correlated with their logP and molecular weight, and metabolites M1 and M2 exhibited higher permeability absorption than ADS-I.

## Conclusion

In this study, the octanol/water distribution coefficients (logP) and membrane permeability properties of ADS-I and its metabolites (M1, M2) were investigated to predict their intestinal absorption in human. Our data suggest that ADS-I has a poor intestinal absorption in human after oral administration. Metabolites (M1, M2) of ADS-I, biotransformed by human intestinal bacteria, exhibited a moderate absorption as well as higher permeability than ADS-I in the following decreasing order: M2 > M1 > ADS-I. These results may suggest that these metabolites may have better absorption than ADS-I, and thus could be the major substances in vivo for inhibitory activities against the growth of tumor after oral administration of ADS-I. In summary, the present results provide useful information to predict the oral bioavailability of ADS-I and its metabolites for the further clinical studies of ADS-I.

## Methods

### Chemicals and reagents

ADS-I and its metabolites M1, M2 (purity > 95 %) were provided by Dr. X.-J. Wang at the Department of Pharmacy, School of Stomatology, the Fourth Military Medical University (Xi’an, China). Ginsenoside Re (purity > 93.7 %) was purchased from the National Institute for Food and Drug Control (Beijing, China), 6-well-Transwell plates (insert diameter 24 mm, pore size 0.4 μm, membrane growth area 4.67 cm^2^) and 96-well plates from Corning Costar (Cambridge, MA, USA), Millicell-ERS system from Millipore Corporation (Bedford, OH, USA), Dulbecco’s Modified Eagle’s medium (DMEM) and fetal bovine serum (FBS) from HyClone Laboratories (Logan, UT, USA), HPLC grade acetonitrile and methanol from Fisher Scientific (Pittsburgh, PA, USA), and Penicillin–streptomycin and 0.25 % trypsin–EDTA solutions from Solarbio (Beijing, China). Other reagents were of analytical purity.

### Determination of Log P of ADS-I metabolites by HPLC–ELSD

#### HPLC–ELSD instrumentation and chromatographic conditions

ADS-I and its metabolites (M1, M2) concentrations in two phases were quantified using a LC-20A high performance liquid chromatograph (Shimadzu Corporation, Kyoto, Japan) equipped with a Alltech type 3300 evaporative light-scattering detector (Alltech Associates, Deerfield, USA). A Diamonsil C_18_ (2) column (4.6 × 250 mm, 5 µm) from Diamonsil Technologies (Beijing, China) was used for all the compound separations, and the column temperature was maintained at 25 °C. The mobile phase consisted of 25 % (A) ultra-pure water and 75 % (B) methanol using an isocratic elution. The flow rate was 1 mL/min, and the injection volume was 10 µl. The ELSD was set to a probe temperature at 60 °C, a gain of 1 and the nebulizer gas nitrogen at a flow of 2.0 L/min.

The liner regression equation for ADS-I was *y* = 1.9726 *x* + 4.6654 (*r* = 0.9995), with a good linearity over the range from 0.1002 to 0.9018 mg/mL, $y = 1.8255x + 4.8093 \, \,( r = 0.9993)$ for M1 with a good linearity over the range from 0.1018 to 0.9162 mg/mL, and $y = 1.8006x + 4.8211\,\,( r = 0.9992)$ for M2 with a good linearity over the range from 0.1010 to 0.9090 mg/mL.

#### LogP of ADS-I and its metabolites with a shake flask method [23, 24]

Prior to the distribution experiment, octanol and phosphate buffer (10 mM, PH 7.4) were mutually saturated at room temperature. ADS-I and its metabolites (M1, M2) were dissolved in DMSO at final concentration of 20 mg/mL, and a volume of 50 µL compound in DMSO solution was added to the octanol/phosphate buffer (1:1, v/v) system. After vortex mixing, the mixtures were orbital shaken for 48 h at 37 °C, and consequently the phases were separated. The solution with two phases were then centrifuged at 13,000 rpm min^−1^ for 10 min. The concentration of ADS-I and metabolites (M1, M2) in both the phosphate buffer and *n*-octanol after the shaking was determined by using HPLC-ELSD as described above.

#### Data analysis

The experiments measured logP was calculated using the following equation: $\log P = \log {{{\text{C}}_{o} } \mathord{\left/ {\vphantom {{{\text{C}}_{o} } {C_{w} }}} \right. \kern-0pt} {C_{w} }}$ where C_o_ was the concentration of a compound in the n-octanol phase, C_w_ is the concentration of the compound in the phosphate buffer phase.

### The permeation characteristics of ADS-I and its metabolites across Caco-2 cell monolayer

#### Cell culture

The human colon Caco-2 cells were purchased from the Cell Bank of the Chinese Academy of Sciences (Shanghai, China), and were cultured in DMEM with 10 % FBS (inactivation at 56 °C for 30 min), 1 % NEAA and 1 % antibiotics (100 IU/ml penicillin and 100 µg/ml streptomycin in a humidified atmosphere of 5 % CO_2_ at 37 °C.

#### Cytotoxicity assay

The cytotoxicity of ADS-I and its metabolites (M1, M2) against Caco-2 cells was evaluated by MTT assay. In brief, 100 µL of Caco-2 cell suspension (2 × 10^4^ cells/mL) per well was seeded in 96-well plates, followed by 24 h incubation (37 °C, 5 % CO_2_). ADS-I and its metabolites (M1, M2) were dissolved in DMSO (<1 ‰) and diluted in DMEM, A volume of 100 µL compounds solution were added to each well and made the final concentrations were 5, 10, 20, 50, 100 µM, respectively. After 4 h incubation, 20 μL of 5 mg/mL MTT was added to each well and incubated for another 4 h. Then the medium was removed and cells were dissolved in 150 µl DMSO with gentle shaking for 10 min, and the optical density (OD) was measured with an ELX800 reader (Bio-Tek instruments, Inc., Winooski, VT, USA) at 490 nm. Untreated cells were used as controls. The doses of a compound with survival rate higher than 90 % were considered non-cytotoxicity.

#### The two-way transport experiment

For transport experiments,Caco-2 cells were seeded on the rat tail collagen-coated 6-wells Transwell plates (insert diameter 24 mm, pore size 0.4 μm, membrane growth area 4.67 cm^2^) at a density of 1 × 10^5^ cells/cm^2^ and incubated for 19–21 days. The medium was changed every 2 days. The integrity and transportation ability of the Caco-2 cell monolayer was examined by measuring the transepithelial electrical resistance (TEER) of filter-grown cell monolayer with millicell-ERS equipment. Only a monolayer with a TEER value of more than 400 Ω cm^2^ was used for the trans epithelial transport experiments.

The transport of ADS-I and its metabolites across Caco-2 monolayer was investigated as previously described [25, 26]. Before the transport experiment, cells were washed three times with warm HBSS (pH 7.4, 37 °C). Cell monolayer was then incubated for 30 min at 37 °C in the transport buffer. To measure the apical (AP)-to-basolated (BL) permeability, 0.5 mL of the transport buffer containing different concentrations (2, 5, 10 µM) of ADS-I, M1, or M2 was added to AP side of the transwell insert, and 1.5 mL of the HBSS was added to the BL chamber. The plates were incubated in an orbital shaker at 37 °C, 50 rpm/min. To assess the drugs transport from AP to BL, after incubation for 30, 60, 90 or 120 min, a volume of 200 μL aliquot was collected from BL side, followed by immediately being replenished with an equal volume of blank HBSS. For the measurement of BL to AP transport, 1.5 mL of the transport buffer containing different concentrations (2, 5, 10 µM) of ADS-I, M1, or M2 was added to the BL side, and 0.5 mL of the HBSS to AP side. A volume of 200 μL aliquot was harvested from AP side at time intervals of 30, 60, 90 and 120 min respectively, and immediately replaced with the same volume of blank HBSS. The samples were frozen immediately and stored at −80 °C before analysis by UHPLC–MS.

In the Caco-2 cell model, the rate of transport was calculated based on the amount transported vs. time curve using linear regression. The apparent permeability (P_app_) presented as an expression of the absorption rate constant was calculated using the following equation,

$${{\rm{P}}_{{\rm{app}}}} = {{{{{\rm{\Delta Q}}} \mathord{\left/ {\vphantom {{{\rm{\Delta Q}}} {{\rm{\Delta t}}}}} \right. \kern-\nulldelimiterspace} {{\rm{\Delta t}}}}} \mathord{\left/ {\vphantom {{{{{\rm{\Delta Q}}} \mathord{\left/ {\vphantom {{{\rm{\Delta Q}}} {{\rm{\Delta t}}}}} \right. \kern-\nulldelimiterspace} {{\rm{\Delta t}}}}} {{\rm{A}} \times {{\rm{C}}_0}}}} \right. \kern-\nulldelimiterspace} {{\rm{A}} \times {{\rm{C}}_0}}}$$

where P_app_ was the apparent permeability coefficient (cm/s), ΔQ/Δt (µmol/s) represented the appearance rate of the test compound on the receiver side, A (4.67 cm^2^) is the surface area of the filter membrane and C_0_ (µmol/L) was the initial concentration in the donor chamber. The efflux ratio (Re) was determined by calculating the ratio of P_app_ (B−A) versus P_app_ (A−B) as the following equation,

$$\text{Re} = {{{\text{P}}_{\text{app}} \left( {{\text{B}}{\mathbf{ - }}{\text{A}}} \right)} \mathord{\left/ {\vphantom {{{\text{P}}_{\text{app}} \left( {{\text{B}}{\mathbf{ - }}{\text{A}}} \right)} {{\text{P}}_{\text{app}} \left( {{\text{A}}{\mathbf{ - }}{\text{B}}} \right)}}} \right. \kern-0pt} {{\text{P}}_{\text{app}} \left( {{\text{A}}{\mathbf{ - }}{\text{B}}} \right)}}$$

#### Sample preparation for UHPLC–ESI–MS/MS assay

A sample (200 μL) from either cellular absorption or transport experiments was mixed with 20 μL methanol containing the internal standard (ginsenoside Re, 0.498 μg/ml). The mixture was vortexed for 60 s. Then after centrifugation at 10,000×*g* for 10 min, 5 μL of supernatant was then injected into the LC/MS system. Also, 200 μL of standard solutions containing different concentrations of ADS-I, M1 or M2 was processed in the same way as above. The calibration curves for ADS-I, M1 or M2 were generated by plotting the peak area ratios of the analytes to the internal standard versus the concentrations by least-square linear regression.

#### UHPLC–MS analysis

UHPLC-MS analysis was performed using an Agilent 1290 Infinity ultra-high performance liquid chromatography (UHPLC) and 6460 type triple quadrupole (QQQ) mass spectrometer equipped with electrospray ion source (ESI) and Mass Hunter working software version B.04.10 (Agilent Technologies, California, USA). A Poroshell 120 EC C_18_ column (2.1 × 100 mm, 2.7 µm) from Agilent Technologies was used as an analytical column and the column temperature was maintained at 25 °C. The isocratic mobile phase consisted of 40 % acetonitrile and 60 % H_2_O at a flow rate of 0.4 mL/min. Quantification was determined using multiple reactions monitoring model, and the operating parameters were optimized as follows: drying gas (N_2_) flow rate, 10.0 L/min; drying gas temperature, 350 °C; nebulizer, 45 psi; capillary, 3500 V; fragmentor voltage, 150 V; sheath gas temperature, 350 °C; sheath gas flow rate, 11 L/min. The precursor-product ion pairs used in MRM mode were: m/z 1073.5 → 927.3 for ADS-I, *m/z* 911.3 → 765.4 for M1, *m/z* 765.4 → 603.1 for M2, 945.5 → 475.3 for Ginsenoside Re as an internal standard.

The sensitivity of UHPLC-MS/MS analysis was first evaluated for drug quantification. The regression equation for the standard curve were as follow: $y = 1.7226x + 0.1239\,( r = 0.9995)$ for ADS-I with the range of 0.061–0.980 μg/mL and the lower limit of quantification was 10 ng/mL; $y = 1.4379x + 0.0417\,( r = 0.9993)$ for M1 with the range of 0.064–1.020 μg/mL and the lower limit of quantification was 8 ng/mL; $y = 1.2802x + 0.0579\,( r = 0.9987)$ for M2 with the range of 0.063–1.000 μg/mL and the lower limit of quantification was 6 ng/mL.

#### Statistical analysis

Data were expressed as means ± standard derivation (SD). Statistical analysis was performed using the statistical software SPSS16.0 (SPSS Inc., Chicago, IL, USA). Student’s *t* test was used to analyze statistical differences between groups. *P* < 0.05 was considered statistically significant.
